# Glycosylation at Asn^211^ Regulates the Activation State of the Discoidin Domain Receptor 1 (DDR1)*[Fn FN2]

**DOI:** 10.1074/jbc.M113.541102

**Published:** 2014-02-07

**Authors:** Hsueh-Liang Fu, Rajeshwari R. Valiathan, Leo Payne, Malika Kumarasiri, Kiran V. Mahasenan, Shahriar Mobashery, Paul Huang, Rafael Fridman

**Affiliations:** From the ‡ Department of Pathology, Wayne State University, Detroit, Michigan 48201,; the ¶ Department of Chemistry and Biochemistry and Walther Cancer Research Center, University of Notre Dame, Notre Dame, Indiana 46556, and; the § Division of Cancer Biology, Institute of Cancer Research, London SW3 6JB, United Kingdom

**Keywords:** Collagen, Glycosylation, Mutagenesis Site-specific, Receptor Regulation, Receptor Tyrosine Kinase

## Abstract

Discoidin domain receptor 1 (DDR1) belongs to a unique family of receptor tyrosine kinases that signal in response to collagens. DDR1 undergoes autophosphorylation in response to collagen binding with a slow and sustained kinetics that is unique among members of the receptor tyrosine kinase family. DDR1 dimerization precedes receptor activation suggesting a structural inhibitory mechanism to prevent unwarranted phosphorylation. However, the mechanism(s) that maintains the autoinhibitory state of the DDR1 dimers is unknown. Here, we report that *N*-glycosylation at the Asn^211^ residue plays a unique role in the control of DDR1 dimerization and autophosphorylation. Using site-directed mutagenesis, we found that mutations that disrupt the conserved ^211^NDS *N*-glycosylation motif, but not other *N*-glycosylation sites (Asn^260^, Asn^371^, and Asn^394^), result in collagen I-independent constitutive phosphorylation. Mass spectrometry revealed that the N211Q mutant undergoes phosphorylation at Tyr^484^, Tyr^520^, Tyr^792^, and Tyr^797^. The N211Q traffics to the cell surface, and its ectodomain displays collagen I binding with an affinity similar to that of the wild-type DDR1 ectodomain. However, unlike the wild-type receptor, the N211Q mutant exhibits enhanced receptor dimerization and sustained activation upon ligand withdrawal. Taken together, these data suggest that *N*-glycosylation at the highly conserved ^211^NDS motif evolved to act as a negative repressor of DDR1 phosphorylation in the absence of ligand. The presence of glycan moieties at that site may help to lock the collagen-binding domain in the inactive state and prevent unwarranted signaling by receptor dimers. These studies provide a novel insight into the structural mechanisms that regulate DDR activation.

## Teaser

**Background:** DDR1 is a receptor tyrosine kinase that signals in response to collagen.

**Results:** Mutagenesis at the ^211^NDS glycosylation site enhances receptor dimerization and results in ligand-independent receptor autophosphorylation.

**Conclusion:** *N*-Glycosylation of DDR1 plays a critical role in maintenance of the inactive state of the receptor dimers.

**Significance:** These studies highlight a new structural feature that regulates DDR1 activation.

## Introduction

**FIGURE 1.**
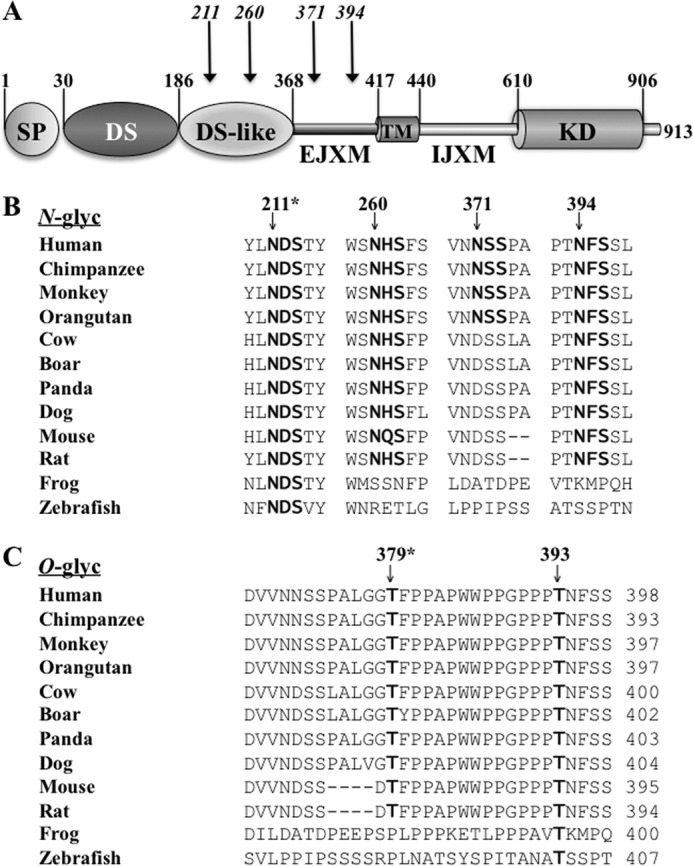
***N*- and *O*-glycosylation sites of DDR1.** *A*, schematic diagram of human DDR1b domain organization. *SP*, signal peptide; *TM,* transmembrane domain. *Numbers* indicate residue number in each domain. *Arrows* show the position of the *N*-glycosylation sites. *B* and *C*, amino acid sequences within the DS-like domain and the EJXM region from various vertebrate species aligned in a Clustal format with the predicted *N*-glycosylation (*N-glyc*) (*B*) and *O*-glycosylation (*O-glyc*) (*C*) sites indicated in *bold. Arrows* indicate the *N*- and *O*-glycosylation sites in the human DDR1b sequence. The *asterisks* in *B* and *C* indicate the amino acid number in the human sequence.

Interactions of cells with the collagenous matrix are mediated by the action of specific cell-surface receptors, which upon activation initiate signaling pathways that alter cell behavior (1, 2). Among the collagen-receptor families, the discoidin domain receptors (DDRs)^3^ are the only receptor tyrosine kinases (RTKs) that undergo autophosphorylation in response to various collagens (1–5). DDRs initiate signaling pathways that are critical for cell-collagen interactions and thus play key roles in many physiological and pathological conditions involving collagen remodeling (summarized in Refs. 1, 3, 6). The DDRs are type I transmembrane glycoproteins, which are represented by two receptors DDR1 and DDR2. The DDR1 subfamily includes five isoforms generated by alternative splicing with DDR1a and DDR1b as the most common isoforms and DDR1d and DDR1e being either truncated or inactive kinases, respectively (5). A single protein represents the DDR2 subtype. DDR1 isoforms undergo autophosphorylation in response to fibrillar and nonfibrillar collagens, and DDR2 is mostly activated by fibrillar collagens (1–5, 7). However, DDR2 can also be activated by the nonfibrillar collagen X (8). Structurally, the ectodomain of DDRs is composed of a discoidin (DS) domain, a DS-like domain, and an extracellular juxtamembrane (EJXM) region, which are followed by a single-pass transmembrane segment (5, 7). The intracellular portion of the receptor is composed of a relatively long intracellular juxtamembrane (IJXM) region and a C-terminal kinase domain (KD) (Fig. 1*A*) (see Refs. 5, 7 for details on domain structure and features).

Besides the fact that DDRs are activated by a unique type of ligand, the collagens, they also display an atypical activation kinetics manifested by a slow and sustained phosphorylation (9), which suggests a unique mechanism of receptor activation that differentiates DDRs from other members of the RTK family (5). Moreover, unlike most RTKs, the DDRs have been reported to form ligand-independent, noncovalent homodimers during biosynthesis, which undergo receptor autophosphorylation upon collagen binding at the cell surface (10, 11). Activation of DDR dimers has been proposed to involve conformational changes induced by binding of collagen to the DS domain but might also involve additional residues within the DS domain that are distal from the collagen-binding site (7, 12, 13). However, the structural rearrangements that lead to dimer autophosphorylation within the KDs, and how collagen binding induces these changes, remain ill-defined. Likewise, it is also unknown how the DDR dimers maintain the autoinhibitory state and whether there are specific structural elements that prevent dimer autophosphorylation. DDRs are glycoproteins containing both *N*- and *O*-glycosylation sites located within the DS-like domain and the EJXM region (5, 7), which have been implicated in receptor stability (14). However, the contribution of glycosylation to receptor activation and collagen binding has not been examined. Here, we report that *N*-glycosylation at the conserved Asn^211^ position plays a key role in the maintenance of the inactive state of the DDR1 dimer.

## EXPERIMENTAL PROCEDURES

### 

#### 

##### Cell Lines

Immortalized monkey kidney COS1 cells (CRL-1650), human embryonic kidney epithelial HEK-293 cells (CRL-1573), and breast cancer HCC1806 cells (CRL-2335) were all obtained from the American Type Culture Collection (Manassas, VA). COS1 and HEK-293 cells were cultured in Dulbecco's modified Eagle's medium, and HCC1806 cells were cultured in RPMI 1640 medium. All media were supplemented with 10% fetal bovine serum (FBS), 2 mm l-glutamine, and antibiotics, and the cells were maintained at 37 °C in an atmosphere of 90% air and 5% CO_2_.

##### Antibodies and Collagen

A rabbit polyclonal antibody (pAb) against the C-terminal region of DDR1 (sc-532) and an antibody against GAPDH (sc-47724) were purchased from Santa Cruz Biotechnology Inc. (Dallas, TX). Goat pAb against the N-terminal region of DDR1 (AF2396) was from R&D Systems (Minneapolis, MN). An anti-phosphotyrosine monoclonal antibody (mAb), clone 4G10®, referred to as anti-Tyr(P) (4G10), was from EMD Millipore (Billerica, MA). A phospho-specific rabbit pAb that recognizes the phosphorylated Tyr^513^ of human DDR1b (Ab92564) (here referred to as Tyr(P)^513^) was purchased from Abcam (Cambridge, MA). The specificity of this antibody for phosphorylated DDR1b, and not for DDR1a, was confirmed in our laboratory (data not shown). Transferrin receptor (TfR) mAb (catalog no. 612124) was from BD Transduction Laboratories (San Jose, CA). A mAb against Myc was a generous gift from Dr. Guri Tzivion (University of Mississippi Medical Center, Jackson, MS). Rat tail collagen type I (catalog no. 3440-100-01) was purchased from Trevigen (Gaithersburg, MD).

##### Sequence Analyses of DDR1

The amino acid sequences of DDR1 used here were as follows: human (*Homo sapiens*), NP_054699.2; chimpanzee (*Pan troglodytes*), NP_001038967.1; monkey (*Macaca mulatta*), AFH28699.1; orangutan (*Pongo abelii*), XP_002816688.1; cow (*Bos taurus*), NP_001069480.2; boar, (*Sus scrofa*), NP_001116577.1; panda (*Ailuropoda melanoleuca*), XP_002928892.1; dog (*Canis lupus familiaris*), XP_003639473.1; mouse (*Mus musculus*), NP_031610.1; rat (*Rattus norvegicus*), NP_001159494.1; frog (*Xenopus laevis*), NP_001083540.1; and zebra fish: (*Danio rerio*), XP_001345829.4. Sequence alignment was generated using ClustalW2. The prediction of *N*-linked and *O*-linked glycosylation sites was performed using NetNGlyc 1.0 Server and NetOGlyc 4.0 Server, respectively.

##### cDNA Constructs, Site-directed Mutagenesis, and Transfections

The DDR1a and DDR1b cDNAs (kindly provided by Dr. Yoshimura, NCI, Frederick, MD) were cloned into the expression vector pcDNA3.1/Myc-His(−)-A (Invitrogen) using conventional cloning approaches. Human DDR1a and DDR1b each harboring a Myc tag at the C-terminal end were constructed as described previously (15). DDR1a and DDR1b harboring substitutions at the *N*- and *O*-glycosylation sites and at other relevant sites (Table 1) were all generated using the QuikChange II site-directed mutagenesis kit (Agilent Technologies, Santa Clara, CA), as described by the manufacturer. The DDR1b ectodomain (ECD) harboring an IgG2 Fc fragment at the C-terminal end was generated by overlapping PCR using the entire ECD of DDR1b (residues Met^1^ to Thr^416^, containing the signal peptide of DDR1b) and the human IgG2 Fc (pFUSE-hFc2-adapt-scFv, a kind gift from Dr. Franck Perez, Curie Institute, Paris, France) cDNAs as templates. The resultant fragments were then cloned into the pcDNA3.1/Myc-His(−)-A expression vector. Amino acid substitutions (N211Q and R105A) in the DDR1b ECD-IgG2 Fc construct were made by site-directed mutagenesis, as described above. The sequences of all wild-type and mutant constructs were verified by DNA sequencing. Expression of the recombinant proteins was accomplished by transient transfection, essentially as we previously described (15).

##### Collagen I-induced DDR1 Activation

Cells were split the day before transfection to 60–70% confluence in 60-mm dishes. The next day, equal amounts of the appropriate expression vectors were transfected using FuGENE 6 (Roche Applied Science) according to the manufacturer's instructions. Four hours after transfection, the medium was aspirated, and the cells were washed twice with PBS, followed by incubation (18 h, 37 °C) in serum-free medium before stimulation. Rat tail collagen type I (10 μg/ml) or vehicle control (20 mm acetic acid) was then added to the serum-free media for 2 h at 37 °C, and then the cell lysates were obtained as described below. In the washout experiments, the collagen-containing media were aspirated after the 2-h incubation time, and the cells were washed (four times) with warm phosphate-buffered saline (PBS) followed by addition of fresh warm serum-free media. At various times (1–24 h), the cells were lysed for analyses. To obtain the cell lysates, the cells were washed twice with cold PBS and then lysed in RIPA buffer (50 mm Tris-HCl, pH 7.4, 150 mm NaCl, 1% Nonidet P-40, 0.5% sodium deoxycholate, and 0.1% SDS) supplemented with protease inhibitors (Roche Applied Science, complete, Mini, EDTA-free), 10 mm NaF, and 1 mm sodium orthovanadate. The cell lysates were cleared by centrifugation at 13,000 × *g* at 4 °C for 10 min; protein concentration was determined using the BCA kit (Thermo Fisher Scientific Inc.), and the lysates were frozen at −80 °C until used.

##### Cell Surface Biotinylation

COS1 cells seeded in 60-mm tissue culture dishes were transfected with wild-type or mutant DDR1 constructs, as described (15). After serum starvation for 18 h, the cells were rinsed with cold PBS/CM (PBS containing 0.1 mm CaCl_2_ and 1 mm MgCl_2_) and then biotinylated with 0.5 mg/ml EZ-link-sulfo-NHS-biotin (Thermo Fisher Scientific Inc.) for 30 min. For control, a parallel plate of cells received PBS/CM without biotin (total lysate). Then the biotin solution was removed, and cells were washed with PBS/CM, and the reaction was stopped by the addition of 50 mm NH_4_Cl for 10 min on ice. The cells were then lysed with RIPA buffer and centrifuged at 13,000 × *g* for 10 min. Equal protein amounts of lysates (400 μg) were then mixed with 120 μl (50% slurry) streptavidin-agarose resin (Thermo Fisher Scientific Inc.) and incubated overnight at 4 °C to capture biotinylated proteins. The mixtures were then briefly centrifuged, and the bound (beads) and unbound (supernatants) fractions were separated and collected. The beads were washed four times with cell harvest buffer (0.5% SDS, 60 mm Tris-HCl, 2 mm EDTA, and 2.5% Triton X-100). The bound biotinylated proteins were then eluted with 2× reducing Laemmli SDS-PAGE sample buffer and boiled. The bound, unbound, and total lysate fractions (40 μg each) were resolved by reducing 8% SDS-PAGE followed by transfer to a nitrocellulose membrane. The blots were probed with anti-Tyr(P) (4G10) antibodies for detection of phosphorylated receptor, and with anti-Myc antibodies for detection of total receptor. The blots were also probed with antibodies to the TfR and GAPDH to evaluate cell-surface proteins and cytosolic proteins level, respectively.

##### Detection of DDR1 Expression and Activation

Lysates of stimulated and unstimulated cells transfected with DDR1 cDNAs were divided into two fractions, and equal amounts of protein from each treatment were resolved by reducing 8% SDS-PAGE followed by immunoblot analyses into two duplicate blots. One blot was probed with anti-Tyr(P) mAb (clone 4G10®) or with anti-Tyr(P)^513^ antibody to DDR1b (Ab92564), and the other blot was probed with anti-Myc antibody. The latter blot was also reprobed with an antibody against GAPDH without stripping.

##### Immunofluorescence Microscopy and Flow Cytometry

COS1 cells grown on 22-mm^2^ glass coverslips were rinsed with PBS, and nonspecific sites were blocked with 0.1% BSA in PBS (1 h, 4 °C). Cells were then incubated (1 h) on ice with 5 μg/ml of the pAb to DDR1 AF2396 diluted in PBS supplemented with 0.1% bovine serum albumin (BSA). After washing with PBS, the cells were fixed with 2% paraformaldehyde in PBS for 15 min at room temperature and washed with PBS supplemented with 0.75% w/v glycine. The cells were then incubated (1 h, room temperature) with a 1:500 dilution of FITC-conjugated donkey anti-goat IgG antibody (Jackson ImmunoResearch) and a 1:5000 dilution of DAPI (Invitrogen) in PBS + 0.1% BSA. Following repeated washes with PBS, the coverslips were mounted on slides with anti-fade reagent (Invitrogen). The samples were examined and photographed using a ×60 oil-immersion objective with a Leica TCS SP5 laser scanning confocal microscope at the Microscopy, Imaging, and Cytometry Resources Core of Wayne State University, School of Medicine. For flow cytometry, COS1 cells transfected with empty vector or vectors containing the WT or the N211Q DDR1b cDNA were harvested using an enzyme-free cell dissociation solution (Millipore) 48 h post-transfection. After washing once with PBS + 1% FBS (wash buffer), the cells were incubated with either purified goat serum or a goat anti-human DDR1 pAb (AF2396, R&D Systems, 10 μg/ml, in PBS + 1% FBS, + 2.5% BSA) for 1 h on ice. Following two washes with wash buffer, the cells were incubated (45 min on ice) with 10 μg/ml donkey anti-goat IgG conjugated with Alexa 488 (Invitrogen). The cells were then washed twice with wash buffer and analyzed for green fluorescence by flow cytometry using an LSR II Analyzer (BD Biosciences). DAPI was added to the cells prior to flow cytometry to discriminate between live and dead cells.

##### Expression and Purification of Recombinant DDR1b ECD-Fc Protein

COS1 cells were seeded in 100-mm dishes transiently transfected with the WT, N211Q, and R105A Fc-tagged DDR1b ECD cDNAs and cultured for 2 days in serum-free media (7 ml/dish). Two days later, the media were collected, and cell debris was pelleted at 3,000 × *g*, and the supernatants (containing the recombinant proteins) were saved for affinity purification using HiTrap Protein G HP Columns (GE Healthcare Life Sciences), according to the manufacturer's instructions. After extensive washes with PBS, the Fc-tagged proteins were eluted with 0.2 m glycine HCl buffer, pH 2.5, followed by neutralization with 1 m Tris-HCl buffer, pH 9.0. Purified proteins were concentrated by a 50-kDa cutoff spin filter (Millipore) and dialyzed against PBS using Slide-A-Lyzer Dialysis Cassettes, 20K MWCO (Thermo Fisher Scientific Inc.). The purity of the recombinant proteins was determined by Coomassie Blue staining after reducing 7.5% SDS-PAGE analysis. Protein concentration of the purified proteins was determined by measuring absorbance at 280 nm and calculated by using the molecular weight of 69,395.53 g/mol (recombinant protein without the signal peptide from DDR1) and an extinction coefficient of 119,480 cm^−1^ m^−1^.

##### Determination of Collagen I Binding Affinity of DDR1 Ectodomain

The relative affinity of the recombinant DDR1 ECD-Fc proteins to immobilized collagen I was determined by ELISA, essentially as described previously (16), but with minor modifications. Briefly, rat tail collagen I was diluted to 10 μg/ml in 10 mm acetic acid before adding to the flat-bottom 96-well plates for an overnight incubation at room temperature. Blocking buffer (0.05 mg/ml κ-casein in PBS supplemented with 0.05% Tween 20) was then added to the wells and the plates were incubated for 1 h at room temperature. Then the blocking buffer was aspirated, and each well was washed once with blocking buffer. The DDR1b ECD-Fc proteins were diluted in blocking buffer at the indicated concentrations and then added to the wells in triplicate. The plates were incubated at room temperature for 3 h, and then the wells were washed with blocking buffer. Goat anti-human IgG Fc antibody conjugated with horseradish peroxidase (Millipore) (1:5000 dilution in blocking buffer) was then added to each well and incubated for 1 h at room temperature. After a final wash, the bound recombinant fusion proteins were detected by adding to each well Substrate Solution (R&D Systems, catalog no. DY999) followed by Stop Solution (2 n H_2_SO_4_) (R&D Systems, catalog no. DY994). The absorbance in each well was measured using a microplate reader set to 450 nm, with a wavelength correction set to 540 nm. The data were fit with GraphPad Prism 5 (GraphPad Software, Inc., La Jolla, CA) using a three-parameter, one-site specific binding model with shared top and bottom values.

##### Chemical Cross-linking of DDR1

Cross-linking of DDR1 in cell lysates was performed essentially as described (17). Briefly, COS1 cells were transfected to express wild-type (WT) or mutant DDR1 constructs, as described (15). After serum starvation overnight, the cells were stimulated with 10 μg/ml rat tail type I collagen for 2 h. A set of parallel dishes was treated with 20 mm acetic acid, as a negative control (unstimulated cells). The cells were then lysed with 20 mm HEPES buffer, pH 7.5, containing 90 mm NaCl, 50 mm NaF, 5 mm Na_4_P_2_O_7_, 1% Triton X-100, 10 mm NaF, and 1 mm sodium orthovanadate, supplemented with protease inhibitors (Roche Applied Science, complete, Mini, EDTA-free). Followed by a brief centrifugation, protein concentration was determined, and aliquots of the lysates were supplemented without or with the amine-to-amine cross-linker BS^3^ (Thermo Fisher Scientific Inc.) (100 and 200 μm, final concentrations) and incubated for 30 min at room temperature. The reaction was quenched with 40 mm Tris, pH 7.5, for 15 min. Then, control and cross-linked lysates (500 μg each) were subjected to an immunoprecipitation procedure by incubating (1 h on ice) the lysates with 0.5 μg/ml anti-DDR1 pAb sc-532. The mixtures were then supplemented with protein A-agarose beads (Thermo Fisher Scientific Inc.) and incubated overnight at 4 °C. The beads were then washed four times with HEPES buffer, and the captured immune complexes were released with 2× reducing Laemmli SDS sample buffer. After a brief centrifugation, the supernatants were boiled and resolved by 6% reducing SDS-PAGE, followed by immunoblot analyses using the goat anti-DDR1 antibody AF2396. The blots were also probed with anti-Tyr(P) mAb 4G10® to detect phosphorylated receptors. The same cross-linking procedure was used to identify endogenous DDR1 complexes in breast cancer HCC1806 cells without and with collagen I stimulation.

##### Reversible Biotinylation Assay

This assay was used to follow the internalization of WT and N211Q DDR1b in unstimulated and stimulated cells. Briefly, serum-starved COS1 cells transfected to express WT or N211Q DDR1b were treated without or with 10 μg/ml collagen I for 30 min at 37 °C. The cells were then washed with PBS/CM and immediately surface-biotinylated on ice with the cleavable sulfo-NHS-SS-biotin reagent for 30 min. Upon completion of the biotin labeling of the surface receptor population and subsequent blocking, warm serum-free media (37 °C) were added to the dishes, which were returned to the 37 °C incubator. At the indicated times (0–40 min), the respective dishes were removed from the incubator and cooled down in ice to halt internalization, and the media were aspirated and the cells washed with cold PBS/CM. Cell surface-bound biotinylating reagent was stripped by incubating the cells twice with 50 mm glutathione in 75 mm NaCl, 10 mm EDTA, pH 7.5, for 15 min per treatment at 4 °C with gentle agitation. After addition of the reducing solution, the cells were washed twice with cold PBS/CM and lysed with RIPA buffer as described above. A plate of biotinylated cells was maintained at 4 °C for 30 min, as a control of total surface DDR1. These plates labeled “TS” did not receive reducing solution; instead, they received PBS/CM. As a negative control, at time 0 min, a plate was treated with glutathione directly after the biotin labeling. The lysates were centrifuged (13,000 × *g*, 20 min), and the supernatants were collected. Protein concentration was then determined in each supernatant using the BCA method. An equal amount of protein from each lysate was then incubated (overnight at 4 °C) with 50 μl of immobilized streptavidin-agarose resin (Thermo Fisher Scientific Inc.) to pull down biotinylated proteins. The beads were washed four times with harvest buffer (0.5% SDS, 60 mm Tris-HCl, 2 mm EDTA, and 2.5% Triton X-100), and the bound biotinylated proteins were eluted with 2× reducing Laemmli SDS sample buffer, boiled, and resolved by 8%, reducing SDS-PAGE followed by immunoblot analyses. The total and endocytosed DDR1 was detected with anti-Myc antibodies. The blots were then reprobed with a mAb to human TfR. Densitometric analysis of the bands of internalized DDR1 in the same blot was performed with the ImageJ program (National Institutes of Health). The density (area per square unit) was plotted against time and expressed in arbitrary units. The relative amount of internalized DDR1b proteins at each time point was calculated as the percentage of biotinylated proteins from the total surface levels of DDR1b (100%).

##### Selective Reaction Monitoring-Mass Spectrometry (SRM-MS)

HEK-293 cells were transiently transfected with WT and N211Q DDR1b cDNA using the calcium phosphate method, as described previously (18). Fifty hours after transfection, the cells were serum-starved overnight and then lysed with a solution of 8 m urea. The lysates were then subjected to reduction, alkylation, and trypsin digestion, as described (18, 19). Briefly, samples were reduced with 10 mm DTT for 1 h at 56 °C and alkylated with 55 mm iodoacetamide for 1 h at room temperature. Samples were digested with 40 μg of trypsin (Promega) overnight at room temperature. Peptides were desalted on a C18 Sep-Pak Plus cartridge (Waters Associates), eluted with 25% acetonitrile, and lyophilized to dryness. Phosphotyrosine-containing peptides were immunoprecipitated from 1.8 mg of lyophilized lysate using 10 μg of pY100 antibody (Cell Signaling Technologies) and 30 μl of protein G beads (Thermo Fisher Scientific Inc.). Immunoprecipitated peptides were eluted in 40 μl of elution buffer (100 mm glycine, pH 2.5), and the beads were removed by centrifugation at 5,000 × *g* for 3 min. Eluted peptides were then transferred to a fresh tube, and 2.5 μl of a heavy peptide standard mix was added per sample to allow for normalization of precipitated endogenous peptide levels between runs. Heavy peptides sequences and amounts are detailed in the supplemental Table 1. Samples were analyzed using a Q-Trap 4000 (ABSciex) mass spectrometer using a previously published procedure (19). Samples containing heavy peptide standards were loaded on a reverse phase (C18) pre-column (100 μm inner diameter, packed with 5–10 cm of 10-μm C18 beads (Millipore)). The pre-column was attached to an analytical column (50 μm inner diameter fused silica capillary packed with 10 cm of 5-μm C18 beads) with an integrated electrospray bottleneck tip with an ∼1-μm orifice. Peptides were eluted using a 75-min gradient with solvent A (acetic acid 1%) and B (H_2_O, acetonitrile, acetic acid at 10:89:1 (v/v/v)) as follows: 10 min from 0 to 10% B, 45 min from 10 to 34% B, 10 min from 34 to 47% B, and 10 min from 47 to 100% B. Transitions were monitored for endogenous and heavy phosphopeptides as detailed in supplemental Table 1. Peak areas were calculated using Analyst version 1.5 software, and peak areas for endogenous peptide transitions were normalized to the corresponding transitions for the spiked heavy peptide standards. Data from the mass spectrometry analyses were expressed relative to total receptor levels, as measured by immunoblot densitometry.

##### Computational Model

We constructed a model of human DDR1 within the plasma membrane environment. The model integrates the discoidin domain that decorates the surface of the membrane to the protein kinase domain that is cytoplasmic, with the two connected via a transmembrane segment. The coordinates of the discoidin domain of DDR1 were obtained from the Protein Data Bank (PDB code 4AG4) (20). The structure shows the glycosylation sites, Asn^211^ and Asn^260^, *N*-linked to chitobiosyl sugar moieties. Superimposing the DDR1 discoidin domain structure with that of DDR2 (PDB code 2WUH) reveals that the structures are highly similar. As a result, we were able to model the triple-helical collagen strands that were shown bound to the structure of DDR2 into the structure of DDR1, which allowed the visualization of collagen binding with respect to the domain and the surface of the membrane. The kinase domain was constructed using its x-ray coordinates (PDB code 4BKJ). For the transmembrane segment, we used secondary structure prediction servers PSIPRED (21) and Jpred3 (22) to predict its secondary structure. Both predicted this segment to be α-helical, which was incorporated into the model. The plasma membrane, which consists of a bilayer of palmitoyloleoylphosphatidylcholine, was modeled using the Desmond System Builder utility implemented in the Maestro graphical interface (version 9.3, Schrödinger LLC, New York). The EJXM and IJXM segments of DDR1 were not modeled because reliable structural homologues for their sequences are lacking.

## RESULTS

### 

#### 

##### Conservation of N- and O-Glycosylation Sites in DDR1 and Their Roles in Receptor Activation

Analyses of the *N*-glycosylation sites in DDR1 reveal two sites located within the DS-like domain at residues 211 and 260 and two sites within the EJXM region at residues 371 and 394 (Fig. 1, *A* and *B*). Crystallographic studies of the DS-like domain of human DDR1 confirmed the presence of *N*-glycosylation at both the Asn^211^ and Asn^260^ positions (20). However, because structural information on the EJXM region is still unavailable, the glycosylation status of Asn^371^ and Asn^394^ remains unknown. Comparison of these *N*-glycosylation sites among vertebrates revealed that the *N*-glycosylation consensus sequence ^211^NDS is the only one that is conserved among the 12 vertebrate species examined (Fig. 1*B*) (20), suggesting that *N*-glycosylation at this specific standard sequon of DDR1 is evolutionarily conserved and thus it may play a key role in receptor regulation. Putative *O*-glycosylation sites in DDR1 are identified at Thr^379^ and Thr^393^ within the EXJM region, with the latter site being conserved throughout evolution (Fig. 1*C*).

##### ^211^NDS N-Glycosylation Motif Regulates the Activation State of DDR1

**FIGURE 2.**
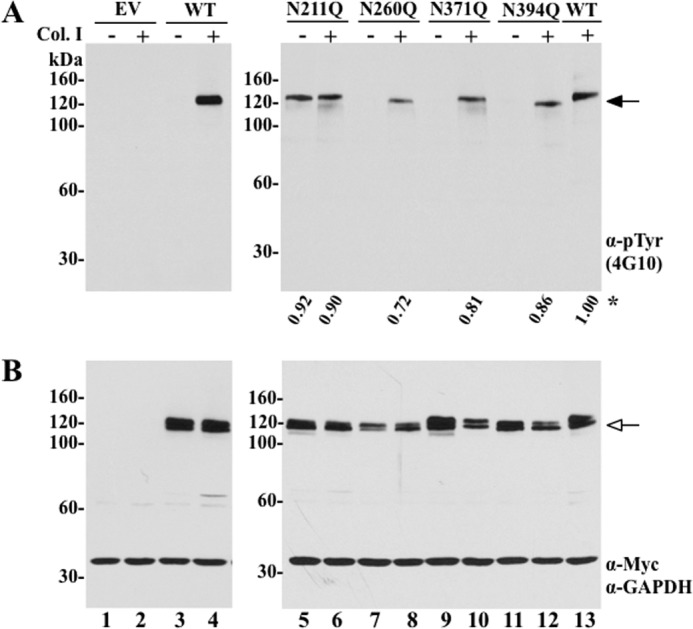
**Collagen I-induced activation of wild-type DDR1b and *N*-glycosylation mutants of DDR1b.** COS1 cells transfected with empty vector (*EV*) or plasmid vectors containing wild-type (*WT*) DDR1b or DDR1b *N*-glycosylation mutant cDNAs were serum-starved (18 h) before stimulation (2 h) with (+) 10 μg/ml rat tail collagen I (*Col. I*) or vehicle control (−), as described under “Experimental Procedures.” After stimulation, the cells were lysed in RIPA buffer, and the lysates from each experimental condition were divided in two fractions. Equal amounts of the two fractions were then resolved by reducing 8% SDS-PAGE in two identical separate gels followed by immunoblot analyses. One blot was probed with anti-Tyr(P) (α-*pTyr*) (4G10®) antibody (*A*) and the other with anti-Myc antibody (*B*). The blot in *B* was then reprobed with antibodies to GAPDH, as loading control. *Black arrow* in *A* indicates phosphorylated DDR1b, and *white arrow* in *B* indicates total DDR1b. *Asterisk* in *A* indicates the level of receptor activation for each mutant as percentage of the activation displayed by WT DDR1b, as determined by densitometry.

To assess the relative contribution of the glycosylation sites in DDR1, we generated receptor mutants by site-directed mutagenesis of the *N*- and *O*-glycosylation sites (listed in Table 1). The wild-type (WT) and mutant DDR1b cDNAs were transiently transfected in COS1 cells for analyses of receptor expression and activation in response to collagen I (15). As a control, COS1 cells were transfected with the pcDNA plasmid without insert (empty vector). Detection of phosphorylated DDR1, under these experimental conditions, can be accomplished directly in cell lysates using anti-Tyr(P) antibodies, without the need of an immunoprecipitation step, as reported previously (15, 23). As shown in Fig. 2, empty vector-transfected COS1 cells show little or no background expression or phosphorylation of endogenous DDR1 under these conditions (Fig. 2, *A* and *B, lanes 1* and *2*), regardless of collagen exposure. In contrast, treatment with collagen I induced a strong DDR1b phosphorylation in COS1 cells expressing WT DDR1b (Fig. 2*A*, *lanes 4* and *13*), which migrates as a species of ∼120–125 kDa. In the absence of collagen, no WT receptor phosphorylation was detected (Fig. 2*A*, *lane 3*). Like WT receptor, the mutants N260Q, N371Q, and N394Q were all phosphorylated in response to collagen I albeit at lower levels (Fig. 2*A*, *lanes 8, 10*, and *12*), whereas none of these mutants were activated in the absence of collagen I (Fig. 2*A*, *lanes 7, 9,* and *11*). Interestingly, we found that a pool of the N211Q DDR1b mutants displayed receptor phosphorylation regardless of collagen I presence (Fig. 2*A*, *lanes 5* and *6*). Moreover, collagen addition did not alter significantly the levels of N211Q constitutive phosphorylation (Fig. 2*A*, *lane 5 versus lane 6*). Similar results were observed with a DDR1a isoform harboring the N211Q substitution (data not shown) and expression of the N211Q mutant in HEK-293 cells (data not shown). Analyses of total cell lysates using anti-Myc antibodies showed that all the DDR1 proteins were detected as doublets, which represent precursor and mature receptor forms (10, 12), migrating as species of ∼110 to ∼120 kDa (Fig. 2*B*). We also noticed that, with the exception of N371Q (Fig. 2*B*, *lanes 9* and *10*), all of the mutants displayed a slight reduction in electrophoretic mobility in reducing SDS gels when compared with WT DDR1b (Fig. 2*B*, *lane 13*), which is consistent with the disruption of *N*-linked glycosylation in the DDR1b mutants. The exception in the case of N371Q may stem from the possibility that the ^371^NSS motif actually is not occupied with glycans (24), or alternatively, the glycosyl moieties linked to this motif do not significantly contribute to the overall mass of DDR1b. DDR1b mutants harboring single or double alanine substitutions at the putative *O*-glycosylation sites, Thr^379^ and Thr^393^, displayed collagen-induced activation. However, the relative levels of receptor phosphorylation were lower than that displayed by WT DDR1 (supplemental Fig. 1), and therefore they were not pursued in this study.

**TABLE 1:** **List of DDR1 mutants**

Mutant name	Substitution	Mutant type
N211Q	Asn^211^ to Gln	*N*-Glycosylation mutant
N211A	Asn^211^ to Ala	*N*-Glycosylation mutant
S213A	Ser^213^ to Ala	*N*-Glycosylation mutant
N260Q	Asn^260^ to Gln	*N*-Glycosylation mutant
N371Q	Asn^371^ to Gln	*N*-Glycosylation mutant
N394Q	Asn^394^ to Gln	*N*-Glycosylation mutant
T379A	Thr^379^ to Ala	*O*-Glycosylation mutant
T393A	Thr^393^ to Ala	*O*-glycosylation mutant
T379A/T393A	Thr^379/393^ each mutated to Ala	*O*-Glycosylation mutant
K655A	Lys^655^ mutated to Ala	Kinase-dead mutant
N211Q/K655A	Asn^211^ to Gln and Lys^655^ to Ala	*N*-Glycosylation and kinase-dead mutant

**FIGURE 3.**
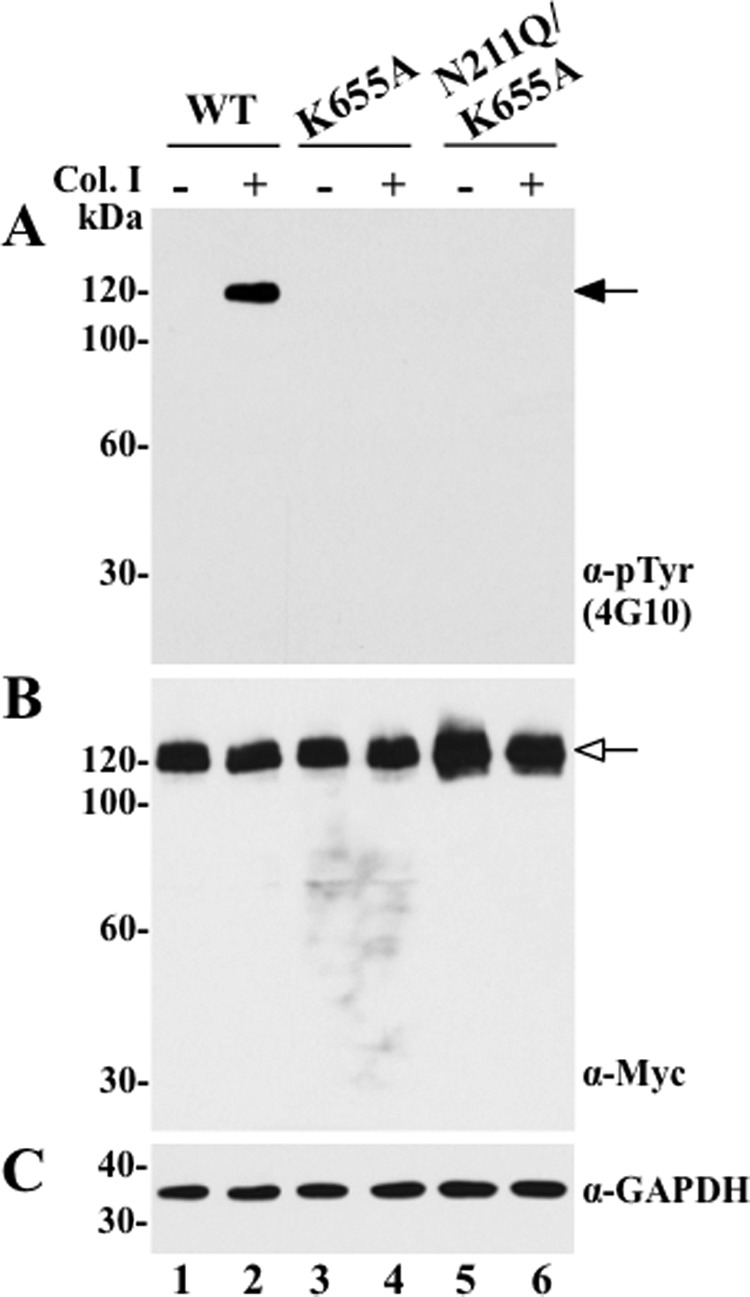
**Intrinsic kinase activity is required for ligand-independent activation of N211Q DDR1b.** COS1 cells expressing wild-type (*WT*), K655A, or N211Q/K655A DDR1b proteins were serum-starved (18 h) before stimulation (2 h) with (+) 10 μg/ml rat tail collagen I (*Col. I*) or vehicle control (−), as described under “Experimental Procedures.” After stimulation, the cells were lysed in RIPA buffer, and the lysates from each experimental condition were divided into two fractions. Equal amounts of the two fractions were then resolved by reducing 8% SDS-PAGE in two identical separate gels followed by immunoblot analyses. One blot was probed with anti-Tyr(P) (α-*pTyr*) (4G10®) antibody (*A*), and the other with anti-Myc antibody (*B*). The blot in *B* was then reprobed with antibodies to GAPDH, as loading control (*C*). *Black arrow* in *A* indicates phosphorylated DDR1b, and *white arrow* in *B* indicates total DDR1b.

To further verify that the ^211^NDS site is important for regulation of DDR1 phosphorylation, and that our observation with a glutamine substitution at the Asn^211^ position is not restricted to the specific nature of glutamine, we generated two additional mutants, N211A and S213A, at the *N*-glycosylation motif ^211^NDS (Table 1). As shown in supplemental Fig. 2*A*, both the N211A and S213A mutants, like N211Q, displayed ligand-independent phosphorylation, which did not significantly change upon collagen stimulation. Thus, the effects observed on DDR1 activation cannot be construed as a nonspecific effect of a single amino acid substitution. To address whether the constitutive activation of the N211Q mutant was due to receptor autophosphorylation or to the action of another kinase(s), we generated a double DDR1b mutant, in which both the Asn^211^ and Lys^655^ residues were substituted for Glu and Ala, respectively, to generate an *N*-glycosylation and a kinase-dead double mutant (N211Q/K655A). The Lys^655^ residue is located within the KD of DDR1, and it is critical for receptor autophosphorylation, when exposed to collagen I (Fig. 3*A*, *lane 4*) (25). Using this double mutant, we found that presence of the K655A mutation in N211Q/K655A, completely abolished the constitutive activation caused by the N211Q substitution (Fig. 3*A*, *lane 5*). This result indicates that the collagen I-independent activation of the N211Q mutant is mediated by receptor autophosphorylation.

To characterize the specific tyrosine residues on the N211Q mutant that are phosphorylated in the absence of collagen I, we employed SRM-MS to quantitatively compare the phosphorylation states of WT and N211Q DDR1b in HEK-293 cells. We have previously employed a similar approach to map the tyrosine phosphorylation profile of DDR2 (19). These analyses showed that the phosphorylation of the N211Q mutant across four tyrosine residues is constitutively up-regulated (greater than 2-fold) compared with wild-type receptor under serum starvation conditions (supplemental Fig. 3*A* and supplemental Table 1). These sites include the highly conserved activation loop phosphorylation sites Tyr^792^ and Tyr^796/797^ as well as IJXM sites Tyr^484^ and Tyr^520^. All four sites that were measured using the SRM-MS assay showed similar fold change increases in phosphorylation *versus* the wild-type receptor suggesting that there is no selective site-specific preference for tyrosine phosphorylation in the N211Q mutant. Note that detection of phosphorylation in WT DDR1b in the absence of collagen by the SRM-MS assay represents background phosphorylation, which is below the detection limit of the immunoblotting technique. In addition, using an antibody recognizing Tyr(P)^513^ (26, 27), a tyrosine residue that is only present in DDR1b, we found that this residue undergoes phosphorylation in WT DDR1b in response to collagen, but it is also constitutively phosphorylated in the N211Q DDR1b mutant upon expression in COS1 cells (shown in supplemental Fig. 3, *B* and *C*). Collectively, these data suggest a key role for *N*-glycosylation at the conserved ^211^NDS site in regulation of the activation state of DDR1.

##### N211Q DDR1b Mutant Displays Sustained Ligand-independent Phosphorylation

**FIGURE 4.**
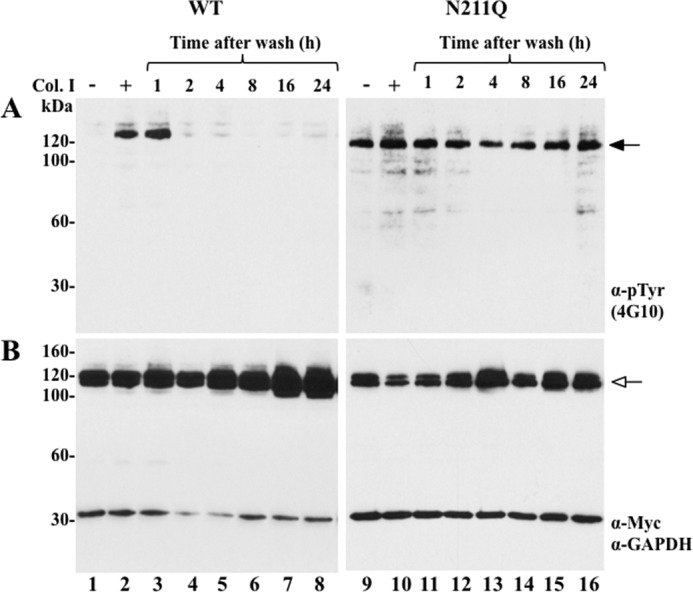
**Sustained activation of N211Q DDR1b.** COS1 cells expressing WT or N211Q DDR1b were serum-starved (18 h) before stimulation (2 h) with (+) 10 μg/ml rat tail collagen I (*Col. I*) or vehicle control (−), as described under “Experimental Procedures.” After stimulation, the media were aspirated, and the cells were washed thoroughly with warm PBS. The dishes were then supplemented with serum-free media and incubated at 37 °C for the indicated times. The cells were lysed with RIPA buffer, and the lysates were analyzed for receptor activation (*A*) and total receptor expression (*B*), as described in Fig. 2. *Black arrow* in *A* indicates phosphorylated DDR1b, and *white arrow* in *B* indicates total DDR1b. Anti-Tyr(P) (α-*pTyr*).

Previous studies demonstrated that DDRs, unlike other RTKs, require relatively long incubations times (∼15–90 min) with collagen to accomplish maximal receptor activation in adherent cells (9, 28, 29). Moreover, the activation (phosphorylated) state of DDRs after collagen stimulation was shown to last for hours (9). On the basis of these observations, the prevailing model of DDR activation postulates that receptor phosphorylation is sustained. However, this model is based on the continuous presence of ligand during the activation period, and therefore, the kinetics of DDR phosphorylation after collagen withdrawal is unknown. Because the N211Q mutant displayed ligand-independent activation, we wished to determine whether the activation of this mutant was also sustained when compared with that of the WT receptor. To this end, we examined the phosphorylation status of WT and N211Q DDR1b as a function of time after withdrawal of ligand. Briefly, serum-starved COS1 cells expressing WT or N211Q were treated with collagen I for 2 h to induce maximal receptor activation followed by extensive washes to remove the collagen. The cells were then incubated for various times (1–24 h) and analyzed for receptor phosphorylation, as described (15). As depicted in Fig. 4, WT DDR1b is activated only when stimulated by collagen I, as expected (Fig. 4*A*, *lanes 1* and *2*). In contrast, N211Q was activated regardless of collagen I stimulation (Fig. 4*A*, *lanes 9* and *10*). Upon withdrawal of collagen, WT DDR1b maintained detectable phosphorylation up to 1 h, after which time receptor activation was no longer detected under these conditions. The N211Q mutant was activated in the absence of collagen (Fig. 4*A*, *lane 9*), and addition of collagen did not significantly alter receptor phosphorylation (Fig. 4*A*, *lane 10*). Interestingly, in contrast to WT DDR1b, the phosphorylated state of the N211Q mutant was readily detected even after 24 h of collagen withdrawal. Taken together, these studies suggest that glycosylation at the Asn^211^ site of DDR1 regulates the temporal dynamics of receptor inactivation, even in the absence of ligand. Moreover, these results also show that ligand withdrawal inactivates the phosphorylation state of WT DDR1 with kinetics inconsistent with sustained activation.

##### Phosphorylated N211Q DDR1b Is Detected Intracellularly and at the Cell Surface

**FIGURE 5.**
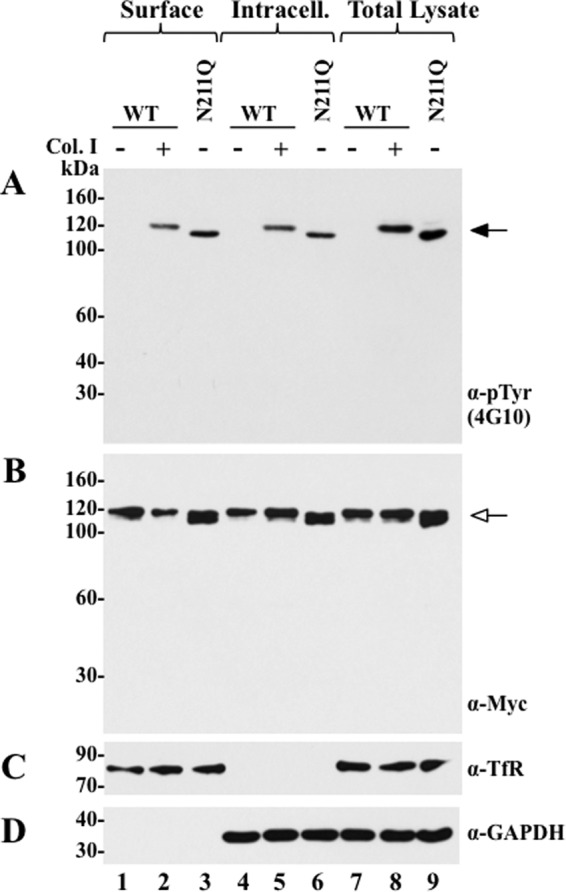
**Subcellular distribution of WT and N211Q DDR1b.** COS1 cells expressing WT DDR1b stimulated (+) or not (−) with collagen I (*Col. I*), and COS1 cells transfected to express the N211Q mutant, which were not stimulated with collagen I, were surface-biotinylated as described under “Experimental Procedures.” The cells were then lysed, and the lysates were incubated with avidin beads to obtain the bound (biotinylated surface proteins) and unbound (nonbiotinylated intracellular proteins) fractions as described under “Experimental Procedures.” Total cell lysates were obtained from the same dishes of biotinylated COS1 cells expressing WT or N211Q DDR1b. Samples (40 μg each) from each fraction were resolved by 8% reducing SDS-PAGE in two identical separate gels followed by immunoblot analysis. One blot was probed with anti-Tyr(P) (α-*pTyr*) (4G10®) antibody (*A*) and the other with anti-Myc antibody (*B*). The blots were reprobed for TfR (85 kDa) (*C*) and GAPDH expression (*D*) using specific antibodies. *Black arrow* in *A* indicates phosphorylated DDR1b, and *white arrow* in *B* indicates DDR1b in each fraction. *Intracell.,* intracellular.

**FIGURE 6.**
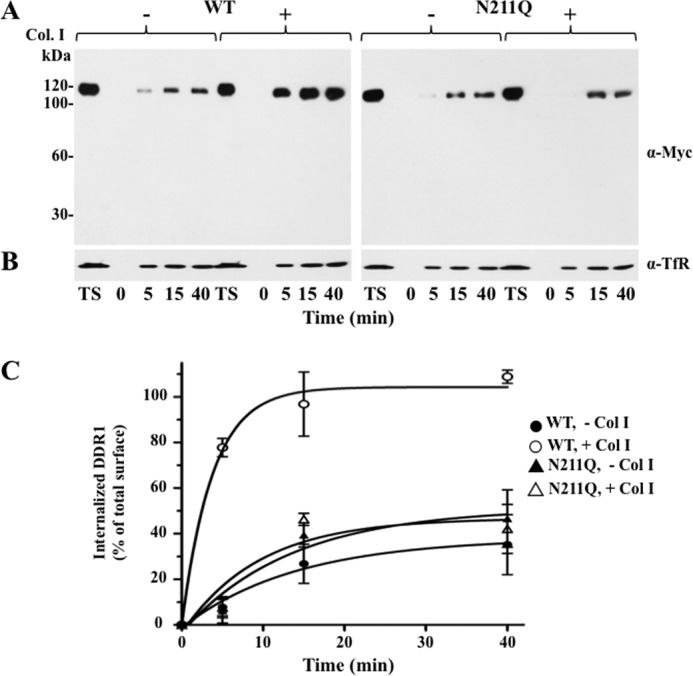
**Endocytosis of WT and N211Q DDR1b.** COS1 cells expressing WT or N211Q DDR1b were serum-starved (18 h) before stimulation (2 h) with (+) 10 μg/ml rat tail collagen I (*Col. I*) or vehicle control (−). Thirty minutes after collagen stimulation, DDR1b internalization in stimulated and unstimulated cells was followed at the indicated times by a reversible biotinylation assay, as described under “Experimental Procedures.” The biotinylated proteins (endocytosed) were resolved by reducing 8% SDS-PAGE followed by immunoblot analyses with anti-Myc (*A*) and anti-TfR (*B*) antibodies. *C*, biotinylated bands of internalized DDR1b proteins were quantified by densitometry, as described under “Experimental Procedures.” *TS,* total surface DDR1b proteins (100%).

The current model of DDR1 activation postulates that DDR1 traffics to the cell surface where it binds to collagen, leading to receptor autophosphorylation (5). However, the N211Q mutant is activated in the absence of collagen. Therefore, we wished to determine the activation status of the N211Q mutant at the intracellular and the cell-surface compartments in the absence of collagen. The subcellular distribution of the N211Q mutant was compared with that displayed by WT DDR1b in cells with or without collagen stimulation. To this end, COS1 cells expressing Myc-tagged WT DDR1b or Myc-tagged N211Q DDR1b mutants were surface-biotinylated, as described under “Experimental Procedures.” Cells expressing WT DDR1b were also stimulated with collagen I. The cells were then lysed, and one aliquot of the lysate was subjected to a pulldown procedure with avidin-conjugated beads to collect the bound (cell-surface proteins) and unbound (nonbiotinylated intracellular proteins) fractions, whereas the second aliquot of the lysate was kept as total cell lysate. The bound, unbound, and total lysate fractions were then subjected to immunoblot analyses using Tyr(P) antibody 4G10 to detect activated receptors, and with an anti-Myc antibody to detect the total pool of receptors. As shown in Fig. 5*A*, stimulation with collagen I resulted in WT DDR1b phosphorylation and distribution of the activated receptor at the cell surface (avidin-bound) and the intracellular compartment (unbound fraction) (Fig. 5*A*, *lanes 2* and *5*, respectively). Intracellular phosphorylated WT DDR1b represents endocytosed receptor (shown also in Fig. 6). Without collagen stimulation, WT DDR1b was not phosphorylated, and consequently no activated WT receptor was detected in any of the subcellular fractions (Fig. 5*A*, *lanes 1, 4,* and *7*). In contrast to WT DDR1b, the N211Q DDR1b mutant displayed phosphorylation in the absence of collagen stimulation, and the autoactivated mutant receptor was readily detected in the avidin-bound fraction (cell-surface proteins) (Fig. 5*A*, *lane 3*), indicating that only the N211Q mutant is phosphorylated at the cell surface in the absence of collagen. Moreover, similar levels of WT and N211Q proteins were detected at the cell surface in the absence of collagen (Fig. 5*B*, *lanes 1* and *3*, respectively). Activated N211Q, but not WT receptor, was also detected in the intracellular fraction in unstimulated cells (Fig. 5*A*, *lane 6*). This indicates that active N211Q DDR1b is also located intracellularly.

Analyses of the total cell lysates (Fig. 5*A*, *lanes 7–9*) (representing input prior to avidin fractionation) with anti-Tyr(P) mAb 4G10® revealed activated receptors only in lysates derived from stimulated cells expressing WT receptor (Fig. 5*A*, *lane 8*) and unstimulated cells expressing N211Q DDR1b (Fig. 5*A*, *lane 9*), as expected. Analyses of the surface, intracellular, and lysate fractions for total receptor expression with the anti-Myc antibody revealed comparable expression levels of WT and mutant DDR1b (Fig. 5*B*). As a control for the subcellular pulldown, the samples were also probed with an antibody to the TfR, a known surface protein, which was readily detected in the avidin-bound fraction (Fig. 5*C*, *lanes 1–3*), as expected, and in the cell lysates (Fig, 5*C, lanes 7–9*). The presence of the N211Q mutant at the cell surface was also detected by immunostaining of nonpermeabilized COS1 cells and by flow cytometry using specific DDR1 antibodies (supplemental Fig. 4, *A* and *B*, respectively). Collectively, these data show that in the absence of collagen stimulation, activated N211Q mutant is detected both intracellularly and at the cell surface. Moreover, the intracellular localization of activated N211Q suggests that the collagen-independent autophosphorylation of N211Q occurs during receptor trafficking to the cell surface.

##### ^211^NDS N-Glycosylation Motif Regulates Collagen I-induced Receptor Endocytosis

Previous studies demonstrated that DDR1 is rapidly internalized after collagen I stimulation (11). In agreement, our subcellular distribution studies indicated that upon collagen stimulation WT DDR1b could be identified in the intracellular compartment (Fig. 5*A*, *lane 5*), consistent with receptor endocytosis (11). Interestingly, activated N211Q was also detected intracellularly. Therefore, we compared the rate of endocytosis of WT and N211Q DDR1b proteins in the presence or absence of collagen I using a reversible biotinylation protocol, as described (30, 31). At various times, internalized biotinylated DDR1b proteins were captured with avidin beads and detected by immunoblot analyses. Fig. 6*A* shows a representative blot depicting the internalized WT and N211Q proteins, and Fig. 6*C* depicts the densitometry analyses of the internalized proteins as a function of time relative to the levels of total surface receptor at the start of the experiment from three independent studies. These data showed a time-dependent endocytosis of WT DDR1b in response to collagen I, consistent with ligand-induced receptor internalization, as described previously (11), and supporting the finding of activated receptor in the intracellular fraction (shown in Fig. 5*A*). In contrast, the N211Q mutant displayed a reduced rate of receptor internalization, which was not altered in the presence of collagen I. Taken together, these data suggest a role for *N*-glycosylation at the Asn^211^ site in regulation of DDR1 internalization. Moreover, the deficient internalization of the N211Q mutant further supports the hypothesis that the intracellular pool of the autophosphorylated mutant (Fig. 5*A*) is due to autoactivation during trafficking to the cell surface.

##### The Ectodomain of the N211Q Mutant of DDR1 Binds Collagen I

**FIGURE 7.**
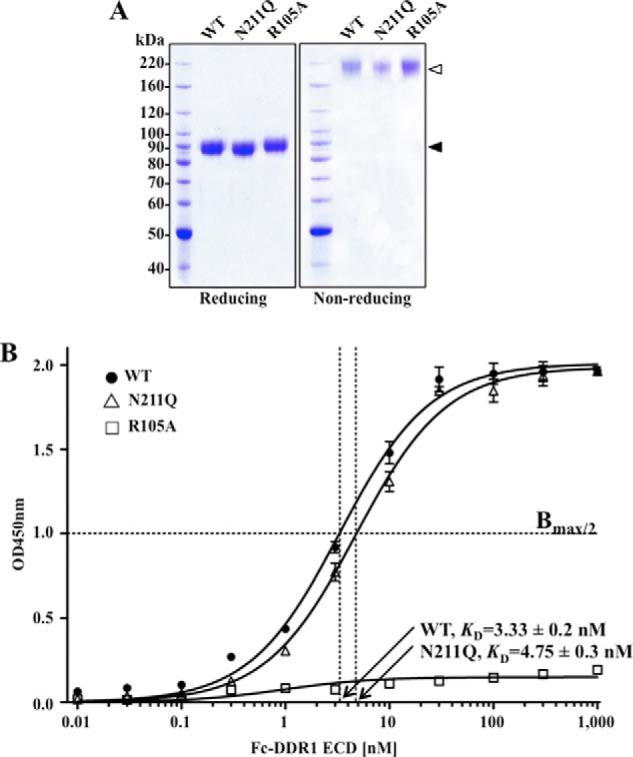
**Collagen I affinity of WT and N211Q DDR1b.** *A*, purified Fc-tagged ECDs of WT, N211Q, and R105A DDR1b (2 μg each) were resolved by 8% SDS-PAGE under reducing and nonreducing conditions, and the gels were stained with Coomassie Blue. *White* and *black arrowheads* indicate the dimeric and monomeric purified proteins, respectively. *B*, various amounts of purified Fc-tagged DDR1b ECDs proteins were added to collagen I pre-coated 96-well plates, and the relative amounts of each protein specifically bound to the coated wells were measured using a microplate reader set to 450 nm, with wavelength correction set to 540 nm, as described under “Experimental Procedures.” The data were fit with GraphPad Prism 5 using a three-parameter, one-site specific binding model with shared top and bottom values. Fc-tagged ECDs of WT (●), N211Q (▵), and R105A (□).

Because the N211Q mutant did not respond to collagen I-induced activation and endocytosis, we asked whether this behavior was due to the inability of the mutant receptor to bind collagen I. Therefore, we measured the relative binding affinities of WT and N211Q DDR1b proteins to collagen I using a solid-phase binding assay. To accomplish this task, we expressed the ECDs of these proteins fused to the human IgG2 Fc fragment at the C-terminal end, as described (16, 23, 32). The presence of the human IgG2 Fc tag promotes ECD homodimerization, which was shown to be required for collagen binding (23). As a negative control, we also expressed an Fc-tagged DDR1b ECD harboring an R105A mutation in the discoidin domain, which was shown to significantly reduce binding of DDR1 to collagen I (13). Indeed, a full-length R105A DDR1b mutant expressed in COS1 cells failed to undergo collagen I-induced receptor phosphorylation when compared with WT DDR1b (supplemental Fig. 5*A*), as reported previously (13). The Fc-tagged ECDs of WT, N211Q, and R105A DDR1b were expressed in COS1 cells and purified to homogeneity (Fig. 7*A*). Because of the presence of the human dimerizing Fc tag, the purified ECDs are able to generate stable SDS-resistant disulfide-linked homodimers, which under nonreducing conditions display a relative mass of ∼180 kDa (Fig. 7*A*, *nonreducing panel*). Binding assays of the ECD homodimers to immobilized collagen I revealed a dose-dependent and saturable binding of WT and N211Q proteins to collagen I with similar affinities (*K_D_* = ∼3–5 nm). In contrast, the R105A mutation abrogated collagen binding, as expected (13), demonstrating the specificity of the interactions (Fig. 7*B*). Taken together, these results suggest that glycosylation at the Asn^211^ site of DDR1 does not significantly contribute to collagen binding, and therefore the inability of the N211Q mutant to undergo collagen I-induced endocytosis cannot be attributed to reduced collagen binding.

##### N211Q Mutant Exhibits Enhanced Dimerization

**FIGURE 8.**
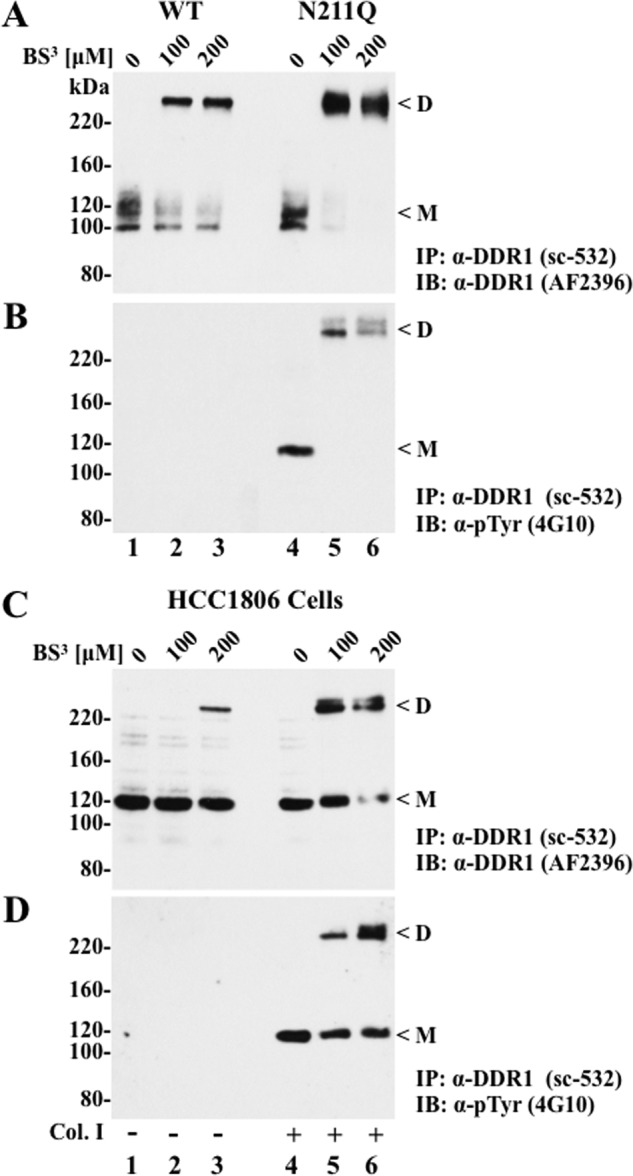
**Cross-linking of WT and N211Q DDR1.** *A–D,* lysates of COS1 cells expressing WT or N211Q DDR1b (*A* and *B*) and lysates of HCC1806 cells treated with (+) or without (−) collagen I (*C* and *D*) were incubated without (0) or with BS^3^ (100 and 200 nm), as described under “Experimental Procedures.” The cross-linked extracts were then subjected to an immunoprecipitation (*IP*) with anti-DDR1 antibody sc-532 and protein A beads. The immunoprecipitates were resolved by reducing 6% SDS-PAGE followed by immunoblot (*IB*) analyses with anti-DDR1 goat pAb AF2396 (*A* and *C*) and anti-Tyr(P) antibody 4G10® (*B* and *D*). *D,* dimeric; *M,* monomeric DDR1 forms.

Previous studies with the epidermal growth factor receptor (EGFR) showed a key role for *N*-glycosylation in regulation of receptor dimerization (33, 34). Therefore, we asked whether the constitutive activation of the N211Q DDR1b mutant was due to regulation of receptor dimerization. To this end, we conducted cross-linking studies in extracts of cells expressing WT or N211Q DDR1b without collagen stimulation, using the chemical cross-linker BS^3^ (10). Because autophosphorylated N211Q was detected intracellularly (shown in Fig. 5*A*) and DDR1 dimerization was reported to occur during receptor trafficking (10), BS^3^ was added after cell lysis, as described (17). Then, the cross-linked extracts were immunoprecipitated with DDR1 antibodies (sc-532), and the resultant precipitates were subjected to immunoblot analyses with the DDR1 ectodomain (AF2396) and Tyr(P) (4G10®) antibodies. As shown in Fig. 8*A*, in the absence of BS^3^, WT and N211Q DDR1b were detected as doublets of ∼100–120 kDa (Fig. 8*A*, *lanes 1* and *4*, respectively). These species represent the monomeric forms under the denaturing and reducing conditions of SDS-PAGE, consistent with the noncovalent nature of DDR1 dimers (10). The cross-linked extracts of cells expressing WT or N211Q DDR1b showed the presence of immunoreactive bands of >220 and ∼110–120 kDa (Fig. 8*A*, *lanes 2* and *3* and *lanes 5* and *6*). These forms are likely to represent dimers and monomers of WT and N211Q DDR1b. Interestingly, relatively higher levels of dimers and reduced levels of monomers were detected in cross-linked N211Q-containing extracts when compared with lysates containing WT receptors (Fig. 8*A*), suggesting increased ligand-independent dimerization in the N211Q mutant. Moreover, only the monomeric (noncross-linked) and dimeric (cross-linked) forms of N211Q, but not of WT DDR1b, were phosphorylated (Fig. 8*B*, *lanes 4–6*), as revealed with the anti-Tyr(P) antibody (4G10). These results indicate that the N211Q dimers are also activated in the absence of ligand. Taken together, these findings suggest a critical role for *N*-glycosylation at the Asn^211^ residue in regulation of DDR1 dimerization.

The detection of cross-linked dimeric DDR1 forms in the COS1 system prompted us to examine the state of DDR1 dimerization in a natural cellular system. HCC1806 is a breast cancer cell line that expresses high levels of endogenous DDR1 (15). Therefore, these cells were used to detect DDR1 dimerization with or without collagen stimulation in cross-linking studies using BS^3^, as described under “Experimental Procedures.” As shown in Fig. 8*C*, addition of BS^3^ (200 μm) to untreated HCC1806 lysates led to the appearance of a DDR1 immunoreactive form of >220 kDa and reduction in the levels of DDR1 monomers (Fig. 8*C*, *lane 3*), consistent with presence of receptor monomers and dimers in unstimulated cells, in agreement with previous studies (10, 11). Collagen I stimulation increased the levels of the >220-kDa forms of DDR1, which were readily detected even at a concentration of BS^3^ of 100 μm (Fig. 8*C*, *lanes 5* and *6*), suggesting that collagen stimulation promotes receptor dimerization. Analyses of receptor phosphorylation demonstrated that both the monomeric and dimeric forms of DDR1 were activated only in the presence of collagen, as expected (Fig. 8*D*, *lanes 4–6*). Hence, collagen stimulation is associated with both increased receptor dimerization and activation.

## DISCUSSION

The DDRs constitute a unique family of RTK because of the nature of their ligands and the kinetics of activation. In addition, DDRs have been shown to exist as inactive kinases while in a dimeric state. However, the mechanism(s) that control the autoinhibitory state of DDR dimers are still unknown. The data presented in this report point to a critical role for *N*-linked glycans at the ^211^NDS sequon in regulation of the autoinhibitory state, dimerization, and internalization of DDR1. The widespread conservation of the ^211^NDS sequon within the DS-like domain of DDR1 throughout evolution and the fact that only alterations at this site disrupted kinase auto-inhibition are consistent with this notion and in agreement with multiple evidence demonstrating a key role for *N*-glycosylation in regulation of RTKs (34–38). Thus, our data add DDR1 to the subgroup of RTKs that are critically regulated by *N*-glycosylation and further highlight the importance of this post-translational modification in receptor function (35, 39). In particular, our data demonstrate a key role for the glycosyl moieties at the Asn^211^ residue in maintenance of the inactive state of the DDR1 dimers. Indeed, mutagenesis at the ^211^NDS conserved site resulted in ligand-independent receptor autophosphorylation, as determined with a DDR1b kinase-dead receptor harboring the N211Q substitution. Our phosphoproteomic analyses revealed that the N211Q mutant becomes phosphorylated at tyrosine residues Tyr^484^ and Tyr^520^ in the IJXM region, and Tyr^792^ and Tyr^796/797^ in the KD when expressed in HEK-293 cells. These residues have been shown to undergo phosphorylation in activated DDR1 and to serve as docking sites for several signaling proteins (40, 41). Interestingly, the finding of phosphorylation at Tyr^796/797^, which are located within the activation loop of the KD, strongly suggests that the active state of the N211Q mutant resembles that exhibited by the activated WT receptor (40).

**FIGURE 9.**
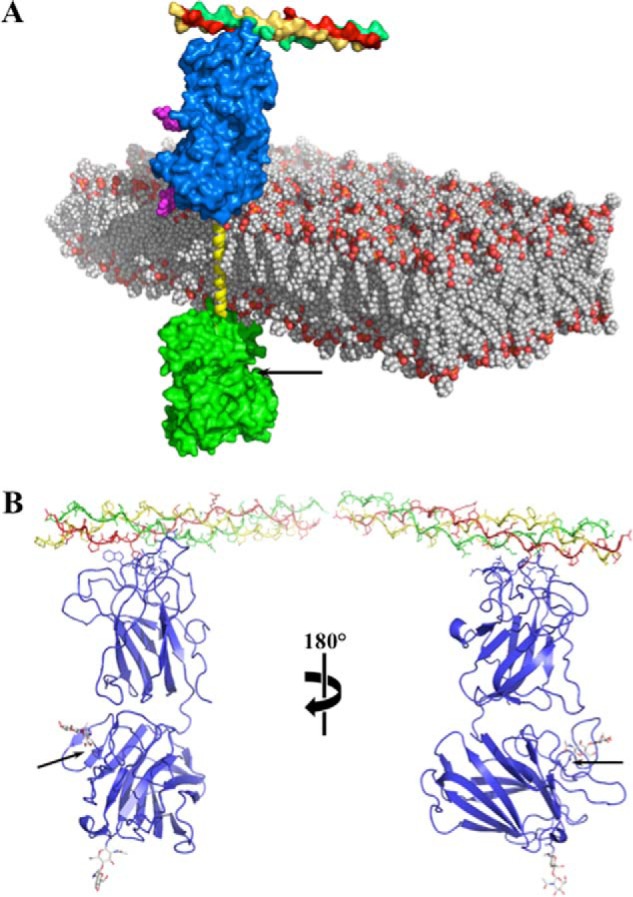
**Schematic of DDR1.** *A*, atomistic model of full-length human DDR1. The KD (PDB code 4BKJ) is in *green* Connolly solvent-accessible surface, and the active site is shown by an *arrow*. The transmembrane segment is shown in *yellow ribbons*, which connects the two domains across the membrane. The discoidin domain (PDB code 4AG4) is shown as a Connolly surface in *blue* with the triple-helical collagen strand bound to it at the distal end to the membrane. Crystallization captured *N*-glycosylation at sites Asn^211^ and Asn^260^ (in *magenta*). The plasma membrane is depicted as a phosphatidylcholine bilayer. *Black arrow* indicates the active site in the KD. *B*, ribbon structure of the DS and DS-like domains of DDR1. The three collagen strands are colored *red, yellow*, and *green*. The backbone of the DS and DS-like domains of DDR1 is depicted as ribbons. The sugar moieties are depicted in a *stick* representation. *Black arrows* indicate the location of the Asn^211^ residue.

Recently, Carafoli *et al.* (20) described the x-ray crystal structure of the DDR1 ECD bound to an antibody Fab fragment. Based on this information, we generated an atomistic model of the entire DDR1 molecule to explain some of the experimental observations (Fig. 9*A*). Collagen-binding site is >80 Å distant from the KD (Fig. 9, *A* and *B*). Transduction of signal would take place via a conformational change produced by the altered dynamics as a result of the first collagen binding event (7). A structural change in the ECD would in turn influence the conformational states of the transmembrane domain helical segment, which itself will propagate to the cytoplasmic end. The glycosyl moiety at Asn^211^ shown in Fig. 9*B* is located within the DS-like domain and is closer to the collagen-binding site at the top of the image, and therefore may participate in regulation of receptor activation. Although the DS-like domain is not directly involved in collagen binding, it has been shown to play a key role in receptor activation because antibodies to this domain blocked collagen-induced DDR1 phosphorylation (20). Thus, it is possible that glycan modifications at the Asn^211^ position, which are close to the DS/DS-like domain interface but do not affect collagen binding, might impose effects (steric or electrostatic) that translate into downstream conformations that counteract the propensity of the transmembrane domain helices of DDR1 to generate active dimers (42) and help to maintain the autoinhibitory state during trafficking of the receptor to the cell surface. In the absence of glycosylation at Asn^211^, the structural transition or dynamic modification would mimic that of the triple-helical collagen bound to the surface domain. As such, the N211Q mutant would appear locked into a conformation that promotes generation of stable receptor dimers and simulates the activated state. Interestingly, the low affinity collagen-binding site in the DS domain faces a protruding loop in the DS-like domain that is very close to Asn^211^ (20). It is conceivable that binding of collagen to this site in WT DDR1 could bypass the structural constraint imposed by the glycosyl moieties in the inactive dimer and lead to enhanced receptor dimerization and activation. The structural studies of Carafoli *et al.* (20) identified a patch of residues at the base of the DS domain that were proposed to serve as a low affinity binding site for collagen and to play a role in the interactions of two DS domains (20). Although this interaction was proposed to be operative within the context of a high affinity collagen-DDR1 dimer complex, it is possible that such interactions might also ensue between DDR1 monomers and collagen. If so, it is tempting to envision a scenario in which an initial low affinity contact between monomeric DDR1 and collagen at the cell surface initiates a series of structural rearrangements that alter the monomer/dimer equilibrium. This may lead to the generation of stable collagen-bound dimers, possibly by promoting transmembrane domain helix-helix interactions (42), resulting in receptor autophosphorylation.

Previously, collagen I binding to DDR1 was shown to induce rapid receptor dimer aggregation and internalization in adherent cells, a series of events that are inconsistent with the reported slow kinetics of receptor activation (11). These findings led the authors to propose a model where DDR1 dimerization, collagen binding, and receptor internalization precede maximal phosphorylation. In agreement with this model (11), we find that DDR1 displays enhanced dimerization and rapid internalization in response to collagen I, consistent with a scenario in which maximal receptor activation, and downstream signaling, occurs within the intracellular milieu. Interestingly, we showed that in contrast to the wild-type receptor a significant proportion of the N211Q constitutively active mutant is present within the intracellular compartment in the absence of collagen. This finding suggests that receptor phosphorylation dictates the localization of the DDR1 protein, favoring the intracellular compartment in its activated form. Furthermore, the cell-surface fraction of N211Q displayed a reduced internalization rate despite being phosphorylated. This feature occurs even in the presence of collagen and could not be ascribed to diminished ligand affinity, as demonstrated by the similar binding affinities of the ECDs of WT and N211Q receptors to immobilized collagen I. This result expands on the previously described model and suggests that receptor phosphorylation and internalization in response to collagen binding are separate events. In EGFR, alterations in glycosylation have also been reported to disrupt receptor internalization (43) indicating that this may represent a common effect of this post-translational modification. Although more studies are required to elucidate the monomer/dimer dynamics of DDR1 receptor pools at the plasma membrane, it is evident that DDR1 exists in both monomeric and dimeric forms in the absence of ligand (our data and Refs. 10, 11), similar to the distribution of EGFR forms (44). It will be interesting to determine whether DDR1 exhibits a mechanism of cytoplasmic driven dimerization analogous to that reported for EGFR, in which a subset of ligand-bound activated receptor dimers promotes phosphorylation and ligand-independent dimerization of neighboring EGFRs (45), and whether this process is regulated by *N*-glycosylation.

It has been shown that both DDR1 and DDR2 display sustained activation after collagen stimulation, which can last for hours (9). This slow dephosphorylation kinetics is considered a distinctive feature of DDR activation, which distinguishes the DDRs from other members of the RTK family (46). It was therefore surprising to find a relatively rapid inactivation of WT DDR1 upon ligand withdrawal under our experimental conditions, in particular when compared with the results of previous studies (9). The mechanisms of this inactivation are still unknown but may proceed through a combination of multiple processes, including internalization and recycling, phosphatase activity, and ubiquitin-mediated receptor degradation (46). Regardless, this finding is, to our knowledge, the first demonstration that ligand availability is required for persistent DDR activation, which reflects the unique characteristic of the DDR ligands. Indeed, the mechanisms of DDR activation and deactivation likely evolved to accommodate the slow turnover of the collagen network and the need for cells to adapt to a unique and mostly steady extracellular signal. However, collagen can also undergo intense turnover and display varying biophysical properties. Thus, it is tempting to speculate that DDRs developed distinct deactivation mechanisms, which provide versatile responses during conditions of fluctuating collagen stability and stiffness as follows: receptor down-regulation upon collagen turnover and sustained active state in steady matrices. Moreover, it also possible that sustained receptor phosphorylation is the result of individual pools of inactive intracellular receptors recycling back to the cell surface and engaging collagen for additional cycles of autophosphorylation. Currently, we have a limited understanding of the regulatory processes involved in DDR trafficking and signaling, and more studies are needed to elucidate the structural features and cellular factors that lead to activation and deactivation of DDRs in various cellular contexts and under various collagen conditions. The studies presented here provide a new insight into the activation of DDR1 and further demonstrate the importance of glycans in regulation of RTKs, which may have implications in diseases with aberrant glycosylation (47).

## Supplementary Material

Supplemental Data

## Footnotes

3 The abbreviations used are:

DDR: discoidin domain receptor
pAb: polyclonal antibody
mAb: monoclonal antibody
DS: discoidin
EJXM: extracellular juxtamembrane
IJXM: intracellular juxtamembrane
KD: kinase domain
ECD: ectodomain
RTK: receptor tyrosine kinase
TfR: transferrin receptor
PDB: Protein Data Bank
EGFR: epidermal growth factor receptor
SRM-MS: selective reaction monitoring-mass spectrometry
BS^3^: bis[sulfosuccinimidyl] suberate.
