# Supplementary material for: Glycosylation at Asn211 Regulates the Activation State of the Discoidin Domain Receptor 1 (DDR1)
Source: J Biol Chem. 2014 Feb 7;289(13):9275–87. doi: 10.1074/jbc.M113.541102 (PMC3979393; doi:10.1074/jbc.M113.541102)
Supplement: Supplemental Data [file supp_289_13_9275__index.html]

Glycosylation at ASN211 regulates the activation state of the discoidin domain receptor 1 (DDR1) — Glycosylation at Asn211 Regulates the Activation State of the Discoidin Domain Receptor 1 (DDR1) — DDR1 Glycosylation and Receptor Activation — Supplemental Data 

# Glycosylation at Asn211 Regulates the Activation State of the Discoidin Domain Receptor 1 (DDR1)

## Supplemental Data

**Files in this Data Supplement:**

- Supplemental Figures (.pdf, 1.5 MB) - Supplemental Figures
