# Supplementary material for: Glycosylation at Asn211 Regulates the Activation State of the Discoidin Domain Receptor 1 (DDR1)
Source: J Biol Chem. 2014 Feb 7;289(13):9275–87. doi: 10.1074/jbc.M113.541102 (PMC3979393; doi:10.1074/jbc.M113.541102)
Supplement: Supplemental Data [file supp_M113.541102_jbc.M113.541102-1.pdf]

Suppl. Fig. 1

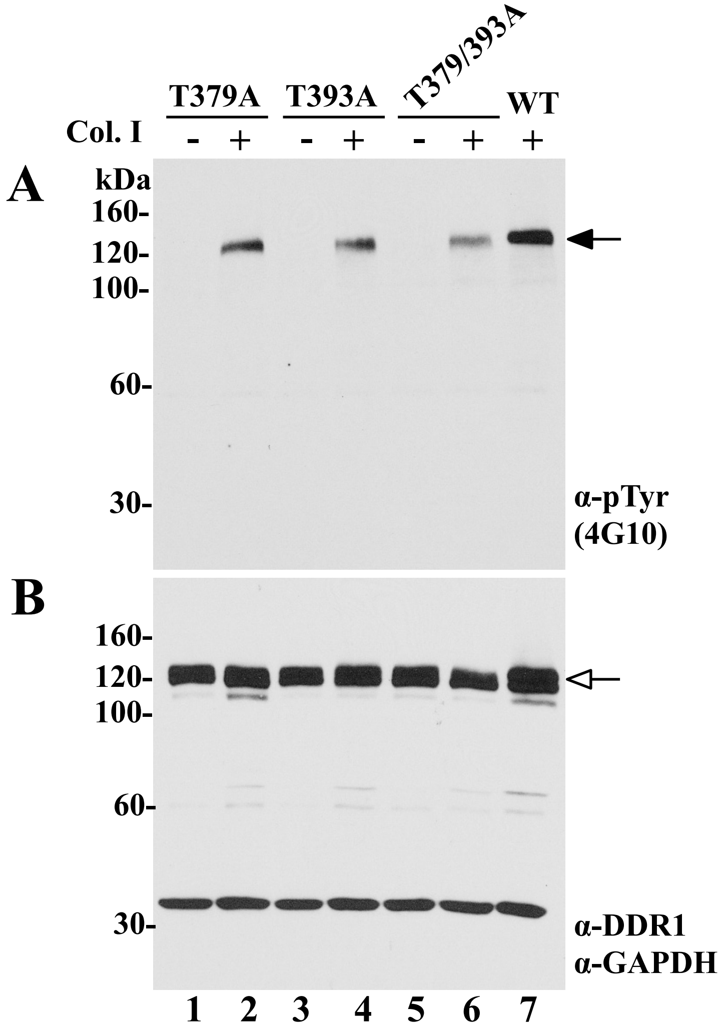

Supplemental Figure 1. Effect of substitutions at *O*-glycosylation sites on DDR1 activation. COS1 cells transfected with plasmid vectors containing wild-type (WT) DDR1b or DDR1b *O*-glycosylation mutant cDNAs were serum-starved (18 h) before stimulation (2 h) with (+) 10 μg/ml of rat tail collagen I (Col. I) or vehicle control (-), as described under "Experimental Procedures". After stimulation, the cells were lysed in RIPA buffer and the lysates from each experimental condition were divided in two fractions. Equal amounts of the two fractions were then resolved by reducing 8% SDS-PAGE in two identical separate gels followed by immunoblot analyses. One blot was probed with anti-pTyr (4G10®) antibody (*A*), and the other with anti-Myc antibody (*B*). The blot in *B* was then reprobed with antibodies to GAPDH, as loading control. Black arrow in *A* indicates phosphorylated DDR1b and white arrow in *B* indicates total DDR1b.

Suppl. Fig. 2

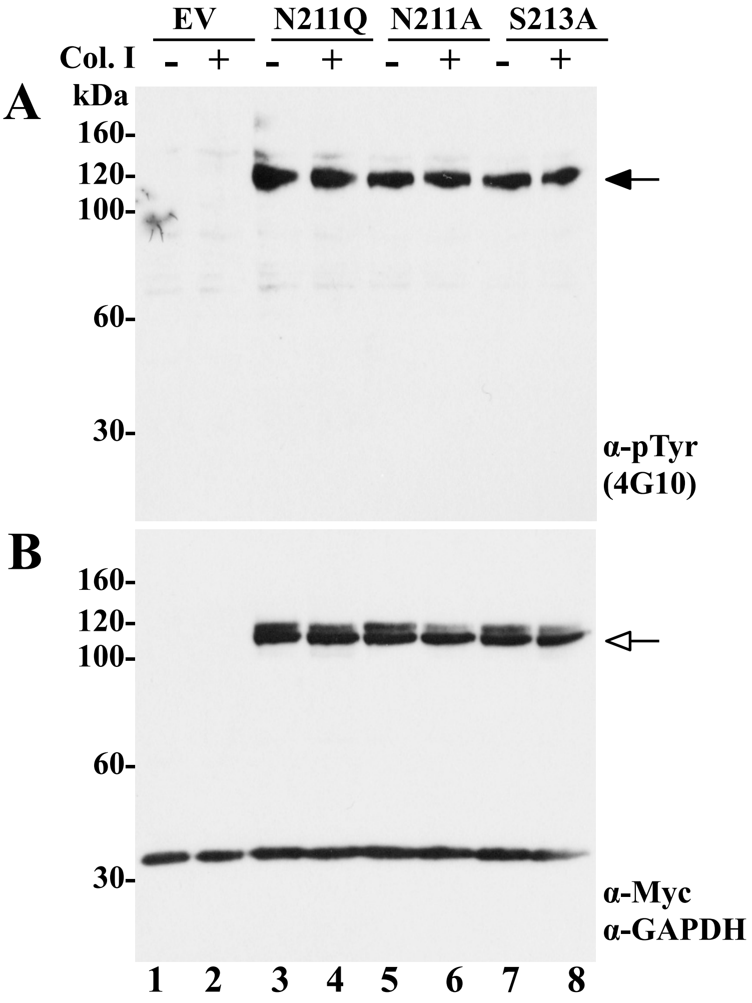

Supplemental Figure 2. **Constitutive activation of DDR1b mutants at the N211 glycosylation sit**e. COS1 cells transfected with plasmid vectors containing wild-type (WT) DDR1b or DDR1b N211 mutant cDNAs were serum-starved (18 h) before stimulation (2 h) with (+) 10 μg/ml of rat tail collagen I (Col. I) or vehicle control (-), as described under "Experimental Procedures". After stimulation, the cells were lysed in RIPA buffer and the lysates were analyzed for receptor activation (*A*) and total receptor expression (*B*), as described in Suppl. Fig. 1. Black arrow in *A* indicates phosphorylated DDR1b and white arrow in *B* indicates total DDR1b.

Suppl. Fig. 3

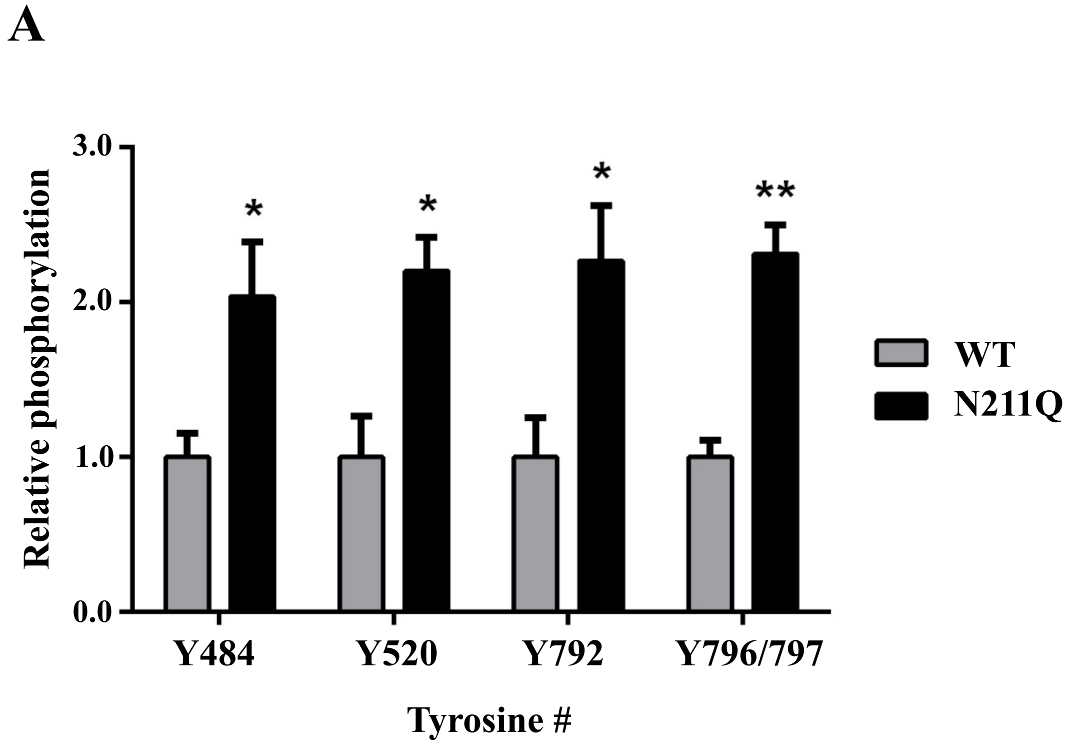

Supplemental Figure 3A. Selective reaction monitoring (SRM) analysis of DDR1b site-specific phosphorylation in wild-type (WT) versus N211➔Q mutant under serum starvation conditions (n=3) expressed in HEK-293 cells. Phosphorylation data is normalised to total receptor levels and expressed as fold change relative to wild-type DDR1. Values are mean ±S.E.M, statistical significance of N211➔Q data compared to WT DDR1b was calculated by unpaired Student's t test where **$p<0.01$ and *$p<0.05$. Representative transitions are shown, as described in Supplemental Table I.

Suppl. Fig. 3, cont'd

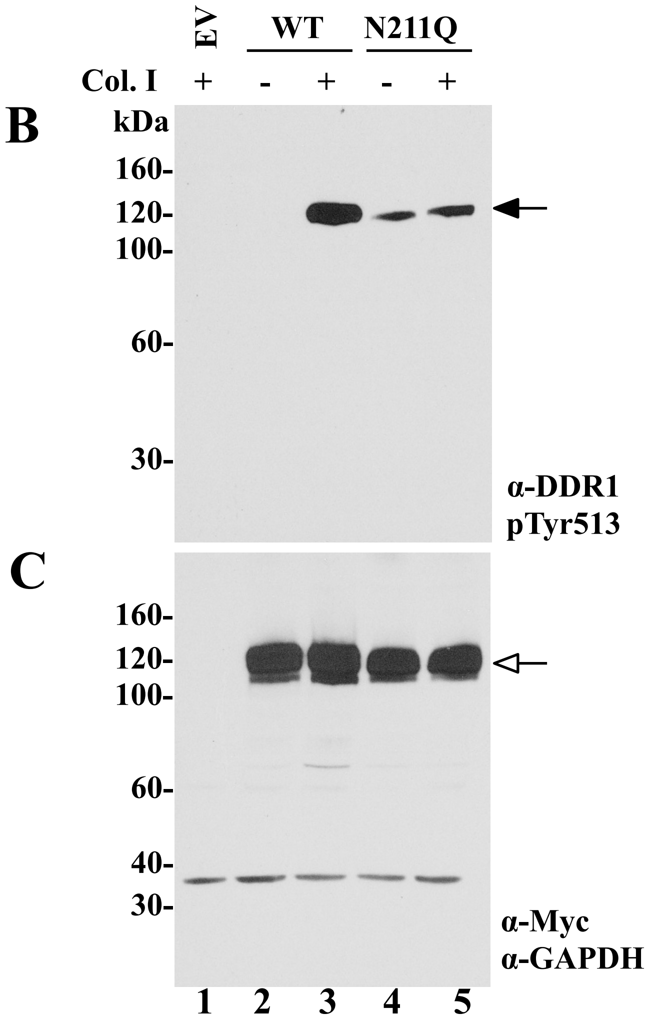

Supplemental Figure 3B and C. WT and N211➔Q DDR1b activation leads to phosphorylation of Y513. COS1 cells transfected with empty vector (EV) or plasmid vectors containing wild-type (WT) or N211➔Q DDR1b cDNA were serum-starved (18 h) before stimulation (2 h) with (+) 10 µg/ml of rat tail collagen I (Col. I) or vehicle control (-), as described under "Experimental Procedures". After stimulation, the cells were lysed in RIPA buffer and the lysates were analyzed for receptor activation (*A*) using a pAb recognizing phosphorylated Tyr513 in human DDR1b (Ab92564). Total receptor expression and equal loading (*B*) were determined using anti-Myc and anti-GAPDH antibodies, respectively. Black arrow in *A* indicates phosphorylated DDR1b and white arrow in *B* indicates total DDR1b.

Suppl. Fig. 4

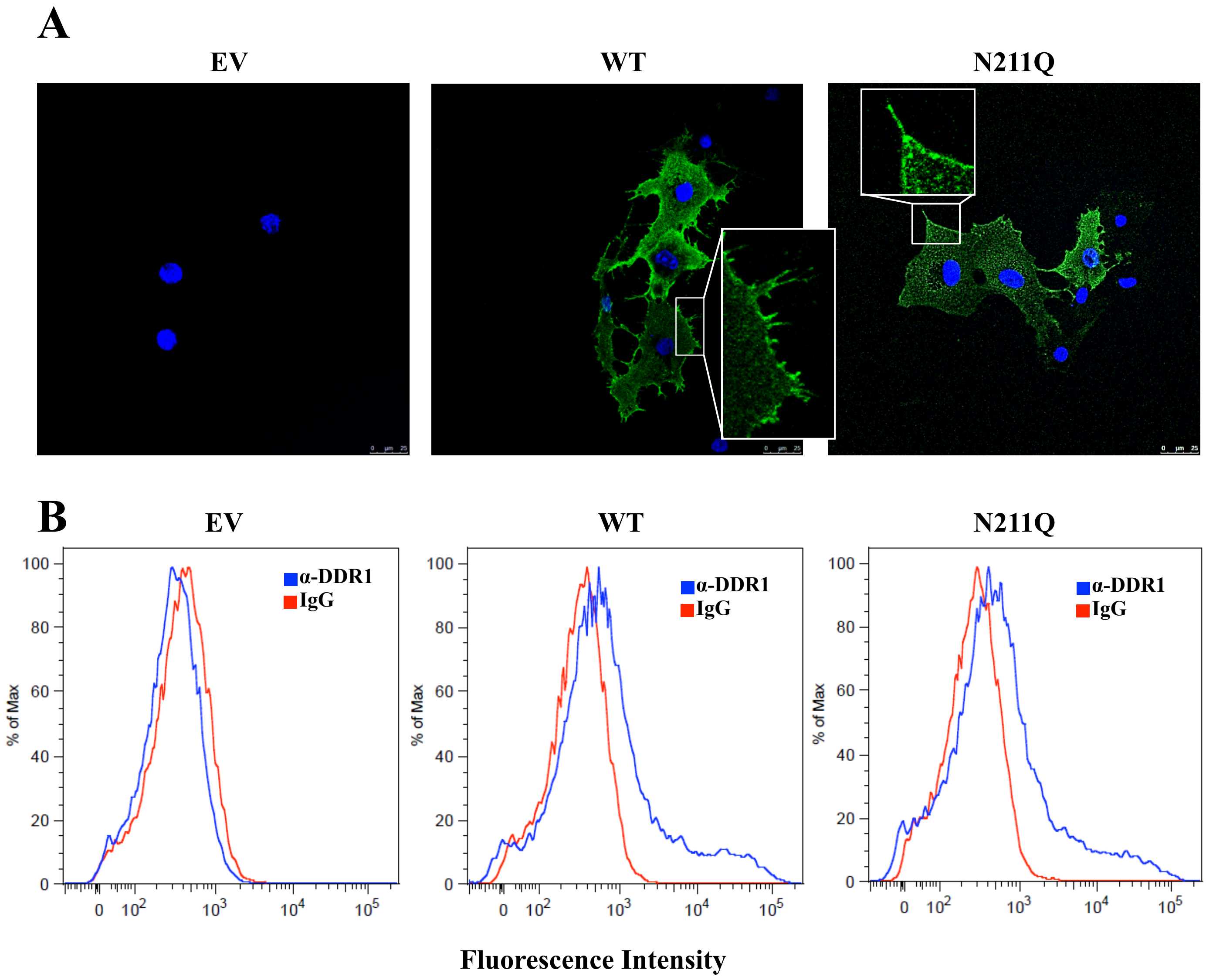

Supplemental Figure 4. Surface localization of WT and N211→Q DDR1b. *A*, COS1 cells transfected with empty vector (EV) or plasmid vectors containing the cDNA of WT or N211→Q DDR1b were seeded on glass coverslips and prepared for immunostaining 72 h post-transfection. The cells were then incubated with pAb to DDR1 and processed for immunofluorescence without permeabilization to label only cell surface DDR1, as described under "Experimental Procedures". White boxes show a magnified view of the cells highlighting the surface localization of the proteins. *B*, The indicated COS1 cells were harvested using an enzyme-free cell dissociation solution 48 h post-transfection and incubated with either goat serum (IgG, red line) or with goat anti-DDR1 pAb (AF2396, blue line) to the ectodomain, on ice, without permeabilization. Alexa-488 conjugated secondary antibodies were used to detect cell surface DDR1 by flow cytometry. All data shown are gated on live, single cells.

Suppl. Fig. 5

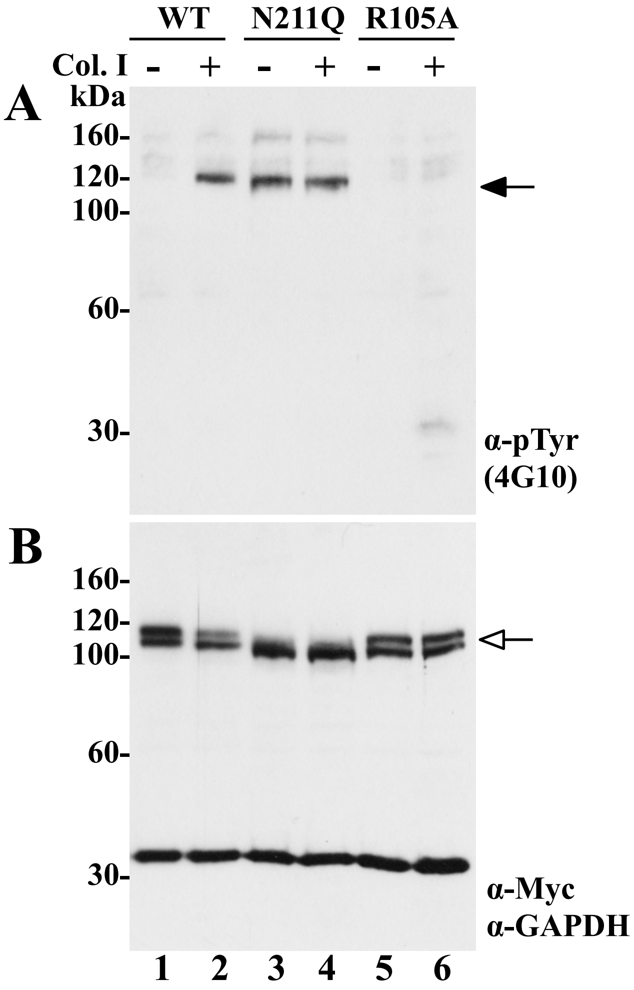

Supplemental Figure 5. **Mutation of R105 inhibits collagen I-induced DDR1b activation**. COS1 cells expressing wild-type (WT) or R105→A DDR1b proteins were serum-starved (18 h) before stimulation (2 h) with (+) 10 µg/ml of rat tail collagen I (Col. I) or vehicle control (-), as described under "Experimental Procedures". After stimulation, the cells were lysed in RIPA buffer and the lysates were analyzed for receptor activation (*A*) and total receptor expression (*B*), as described in Suppl. Fig. 1. Black arrow in *A* indicates phosphorylated DDR1b and white arrow in *B* indicates total DDR1b.

Suppl. Table I

Supplemental Table I.
*Transitions Used for the SRM-MS Assay**

Phosphosite	Peptide	Amount loaded per run (pmol)	Transition (Q1/Q3)	Fragment ID	Collision energy (V)
DDR1 Y484 (heavy)	EPPP[Y(PO3H2)]QEPRP[Arg(13C6;15N4)]	6.2	**728.27/535.33**	y4	42.5
			728.27/282.18	y2	42.5
			728.27/1132.52	y8	42.5
			728.27/1229.57	y9	42.5
DDR1 Y520 (heavy)	LLLAT[Y(PO3H2)]ARPP[Arg(13C6;15N4)]	6.2	**680.81/227.18**	y2	39.5
			680.81/379.23	y3	39.5
			680.81/950.45	y7	39.5
			680.81/1021.49	y8	39.5
DDR1 Y792 (heavy)	NL[Y(PO3H2)]AGDYY[Arg(13C6;15N4)]	3.1	**612.75/511.25**	y3	35.0
			612.75/683.29	y5	35.0
			612.75/754.33	y6	35.0
			612.75/997.36	y7	35.0
DDR1 Y796 (heavy)	NLYAGD[Y(PO3H2)]Y[Arg(13C6;15N4)]	3.1	**612.75/763.26**	y5	35.0
			612.75/834.29	y6	35.0
			612.75/997.36	y7	35.0
DDR1 Y484 (endogenous)	EPPP[Y(PO3H2)]QEPRPR	N/A	**723.27/525.33**	y4	42.5
			723.27/272.18	y2	42.5
			723.27/1122.52	y8	42.5
			723.27/1219.57	y9	42.5
DDR1 Y520 (endogenous)	LLLAT[Y(PO3H2)]ARPPR	N/A	**675.81/227.18**	y2	39.5
			675.81/369.23	y3	39.5
			680.81/940.45	y7	39.5
			680.81/1011.49	y8	39.5
DDR1 Y792 (endogenous)	NL[Y(PO3H2)]AGDYYR	N/A	**607.75/501.25**	y3	35.0
			607.75/673.29	y5	35.0
			607.75/744.33	y6	35.0
			607.75/987.36	y7	35.0
DDR1 Y796 (endogenous)	NLYAGD[Y(PO3H2)]YR	N/A	**607.75/753.26**	y5	35.0
			607.75/824.29	y6	35.0
			607.75/987.36	y7	35.0

* Values in bold indicate representative transitions used in Supplemental Figure 3.
